# Prediction of overall survival for metastatic pancreatic cancer: Development and validation of a prognostic nomogram with data from open clinical trial and real‐world study

**DOI:** 10.1002/cam4.1573

**Published:** 2018-06-01

**Authors:** Junjie Hang, Lixia Wu, Lina Zhu, Zhiqiang Sun, Ge Wang, Jingjing Pan, Suhua Zheng, Kequn Xu, Jiadi Du, Hua Jiang

**Affiliations:** ^1^ Department of Oncology Changzhou No.2 People's Hospital the Affiliated Hospital of Nanjing Medical University Changzhou China; ^2^ Department of Oncology Shanghai JingAn District ZhaBei Central Hospital Shanghai China; ^3^ Center of Data Mining and Business Analytics Rutgers Business School Newark NJ USA

**Keywords:** metastatic pancreatic cancer, open clinical trial, overall survival, prognostic nomogram, real‐world study

## Abstract

It is necessary to develop prognostic tools of metastatic pancreatic cancer (MPC) for optimizing therapeutic strategies. Thus, we tried to develop and validate a prognostic nomogram of MPC. Data from 3 clinical trials (NCT00844649, NCT01124786, and NCT00574275) and 133 Chinese MPC patients were used for analysis. The former 2 trials were taken as the training cohort while NCT00574275 was used as the validation cohort. In addition, 133 MPC patients treated in China were taken as the testing cohort. Cox regression model was used to investigate prognostic factors in the training cohort. With these factors, we established a nomogram and verified it by Harrell's concordance index (*C*‐index) and calibration plots. Furthermore, the nomogram was externally validated in the validation cohort and testing cohort. In the training cohort (n = 445), performance status, liver metastasis, Carbohydrate antigen 19‐9 (CA19‐9) log‐value, absolute neutrophil count (ANC), and albumin were independent prognostic factors for overall survival (OS). A nomogram was established with these factors to predict OS and survival probabilities. The nomogram showed an acceptable discrimination ability (*C*‐index: .683) and good calibration, and was further externally validated in the validation cohort (n = 273, *C*‐index: .699) and testing cohort (n = 133, *C*‐index: .653).The nomogram total points (NTP) had the potential to stratify patients into 3‐risk groups with median OS of 11.7, 7.0 and 3.7 months (*P* < .001), respectively. In conclusion, the prognostic nomogram with NTP can predict OS for patients with MPC with considerable accuracy.

## 1 INTRODUCTION

Pancreatic cancer is the 7th leading cause of cancer‐related mortality worldwide and the 6th one in China.^1^, ^2^ At the time of initial diagnosis, most patients lose the chance of resection.^3^ As a consequence, the 5‐year relative survival rate of patients with pancreatic cancer remains as low as 6%.^4^ Although recently developed regimens of chemotherapy significantly improved the prognosis of advanced pancreatic cancer, only a few strictly selected patients obtained the benefit.^5^ Thus, it is necessary to search for reliable prognostic tools for overall survival (OS) providing valuable information for precise decision‐making at the time of diagnosis.^6^

Up to date, various kinds of prognostic models were reported in pancreatic cancer (Table S1).^7^, ^8^, ^9^, ^10^, ^11^, ^12^, ^13^, ^14^, ^15^ Overall, there were 3 common types of models: nomogram, models based on the regression coefficient of prognostic factors and models based on the number of prognostic factors. It seems that only nomogram can provide a valid clinical outcome for each patient and is gradually used in evaluating cancer prognosis.^16^ A nomogram is a simple graphical tool integrating diverse prognostic variables and can meet our requirements for the personalized medicine.^17^ The most widely used prognostic factors in these models included age, sex, tumor size, performance status, regional lymph node metastasis, distant metastasis, carbohydrate antigen 19‐9 (CA19‐9), and back pain. Moreover, several nomograms were constructed in pancreatic cancer with different stages.^8^, ^9^, ^10^, ^11^ However, most of them were based on retrospective data and none of them mainly focused on metastatic pancreatic cancer (MPC). In one of these studies, there was a large heterogeneity among the enrolled 327 MPC patients with first‐line chemotherapy, leading to an inevitable bias.^10^

The aim of this study was to develop and validate a nomogram using baseline clinicopathological variables to predict OS of patients with MPC. This model was first developed based on data of the comparator arm in 2 trials (the trial numbers: NCT00844649 and NCT01124786), and then validated externally in another trial (the trial number:NCT00574275) and real‐world study.

## 2 METHODS

### 2.1 Trial selection

Three trial datasets (the comparator arm only) were selected in the Project Data Sphere (PDS), a not‐for‐profit initiative allowing collective historical cancer clinical trial data to be shared in public.^18^ Among them, 2 trials of NCT00844649 and NCT01124786 were the source of the training cohort while the trial of NCT00574275 was taken as the validation cohort. NCT00844649 was a randomized Phase III trial comparing OS and progression‐free survival (PFS) between combination of ABI‐007 (Albumin‐bound Paclitaxel) with gemcitabine and gemcitabine monotherapy in patients diagnosed with metastatic pancreatic adenocarcinoma.^19^ NCT01124786 was a randomized phase II multicenter trial comparing CO‐1.01 with gemcitabine as first‐line therapy in patients with metastatic pancreatic adenocarcinoma.^20^ NCT00574275 was a multinational, randomized, phase III double‐blind trial comparing the efficacy of aflibercept vs the placebo in patients treated with gemcitabine for MPC.^21^

### 2.2 Patients

In the 3 trials, the comparator arm was composed of MPC patients treated with gemcitabine as first‐line chemotherapy and the primary end point was OS. Furthermore, there were some similarities in both inclusion and exclusion criteria: above 18 years of age, histological or cytological confirmation of metastatic adenocarcinoma of the pancreas, adequate renal, liver, and bone marrow functions, a Karnofsky performance status (KPS) ≥70 or Eastern Cooperative Oncology Group (ECOG) performance status ≥2. The datasets for each individual were de‐identified when acquired from PDS. For the training cohort, we selected patients with available demographic information and clinicopathological characteristics. Also, we required complete information of prognostic factors identified in the training cohort from all patients included in the validation cohort. In total, 445 patients were included in the training cohort and 273 subjects in the validation cohort. In addition, 133 MPC patients treated in Changzhou No 2 People's Hospital and Shanghai JingAn District ZhaBei Central Hospital were enrolled in this study. The inclusion and exclusion criteria were: (1) histological or cytological confirmation of metastatic adenocarcinoma of the pancreas; (2) ECOG performance status ≥2; (3) above 18 years of age; (4) without any concurrent cancer at another organ site.

### 2.3 Statistical analysis

The statistical analyses were performed with R 3.3.1 software (Institute for Statistics and Mathematics, Vienna, Austria) and SPSS statistical software (version 21.0; SPSS Inc, IBM, Armonk, NY, USA). For patients enrolled in the clinical trial, OS was calculated from the date of randomization to death due to any cause or censored at the last known time the participants were alive. Cox regression model was used to investigate independent prognostic factors for OS. Given the prognostic value and β‐coefficient of these factors, performance status, liver metastasis, CA19‐9 log‐value, absolute neutrophil count (ANC), and albumin were further chosen to establish a nomogram to predict median OS time and survival probabilities at 3‐, 6‐, 9‐, and 12‐months. The internal validation of this nomogram contained 2 components. First, the *C*‐index was used to assess the predictive accuracy of the nomogram. The second validation component was the calibration plot comparing the predicted survival probability with the actual survival. The internal validation of the nomogram was performed using bootstrapping with 1000 re‐samples. To give a reasonable discrimination of OS, 2‐ or 3‐risk groups were built based on optimal cut‐off values of the nomogram total points (NTP) identified by the Elucidian method^22^ or Cox's method, respectively. The Kaplan‐Meier analysis was performed to show the stratification of OS in different risk groups. To assess the external applicability of the nomogram, the results were further confirmed in the validation cohort and testing cohort. Two‐sided *P* < .05 was considered statistically significant in all tests.

## 3 RESULTS

### 3.1 Patient characteristics

**Figure 1:**
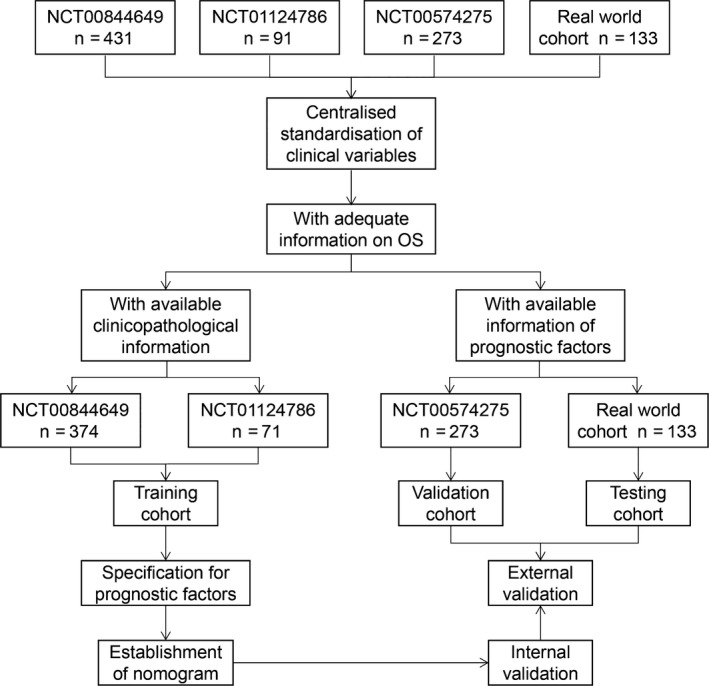
Study design

The overall study design is shown in Figure 1. Table 1 demonstrated the baseline clinicopathological characteristics of patients with MPC in 3 clinical trials and real‐world cohort. The training dataset included 374 participants from NCT00844649 with a median survival time of 6.7 months and 71 patients from NCT01124786 with a median survival time of 6.0 months. The validation dataset was composed of 273 patients from NCT00574275 with a median survival time of 7.8 months. The testing cohort consisted of 133 patients from real‐world study with a median survival time of 6.1 months.

**Table 1:** Baseline clinicopathological characteristics of patients with MPC

	Training cohort 1‐NCT00844649 (n = 374)	Training cohort 2‐NCT01124786 (n = 71)	Validation cohort‐ NCT00574275 (n = 273)	Testing cohort (n = 133)
Age (y, range)	63 (32‐88)	62 (40‐79)	61 (34‐86)	62 (34‐83)
Gender				
Male	224	45	156	85
Female	150	26	117	48
Performance status				
ECOG PS = 0 (or KPS = 100)	61	16	98	11
ECOG PS = 1 (or KPS = 90 or 80)	289	55	159	85
ECOG PS = 2 (or KPS = 70)	24	0	16	37
Body mass index (kg/m^2^)				
Normal weight (18.5‐25)	168	45	146	97
Underweight <18.5	13	2	16	26
Overweight (25‐30)	135	18	80	10
Obese ≥30	58	6	31	0
Primary tumor site				
Head and neck	155	0	115	47
Body and tail	218	0	86	86
Entile pancreas	0	0	72	0
Unknown	1	71	0	0
Site of metastatic disease				
Liver	308	59	214	106
Lung	184	24	67	41
CA19‐9 (U/mL)	2554.4 (0.3‐12 207 654.2)	3850.0 (3.0‐798 000.0)	1080.0 (0.6‐1 743 408.0)	902.0 (0.6‐2084.0)
Missing	32	0	14	0
RBC (×10^12^/L)	4.25 (3.01‐5.64)	4.30 (2.90‐5.68)		3.97 (1.24‐5.4)
Missing	10	0	273	0
Hemoglobin (g/L)	127 (91‐169)	124 (82‐159)	127 (75‐167)	120 (71‐157)
Missing	10	0	2	0
WBC (×10^9^/L)	7.9 (2.8‐23.1)	7.9 (4.3‐25.4)	7.8 (3.4‐38.6)	6.6 (2.7‐31.5)
Missing	10	0	2	0
ALC (×10^9^/L)	1.4 (0.3‐4.0)	1.4 (0.4‐5.4)		1.3 (0.4‐4.9)
Missing	15	0	273	0
ANC (×10^9^/L)	5.6 (1.5‐18.2)	5.6 (2.5‐22.6)	5.4 (1.6‐35.8)	4.3 (1.2‐26.3)
Missing	11	0	4	0
AMC (×10^9^/L)	0.5 (0‐3.2)	0.4 (0‐1.3)		0.5 (0‐3.5)
Missing	12	0	273	0
PLT (×10^9^/L)	234 (88‐632)	273 (85‐620)	255 (88‐872)	177 (37‐763)
Missing	13	0	2	0
Albumin (g/L)	42 (28‐53)	41 (23‐51)	40 (25‐62)	39 (26‐48)
Missing	10	0	14	0
Protein (g/L)	71 (51‐90)	68 (52‐80)	72 (57‐92)	67 (51‐86)
Missing	10	0	8	0
ALT (IU/L)	31 (8‐209)	30 (10‐270)	33 (1‐159)	22 (4‐190)
Missing	10	0	3	0
AST (IU/L)	28 (9‐119)	27 (13‐165)	29 (1‐104)	26 (11‐156)
Missing	10	0	2	0
ALP (U/L)	135 (40‐1086)	158 (51‐1963)	163 (50‐2331)	120 (36‐1185)
Missing	10	0	2	6
LDH (U/L)	196 (99‐1133)	178 (96‐964)		180 (70‐1050)
Missing	14	0	273	0
Bilirubin (μmol/L)	8.6 (1.7‐35.9)	8.6 (3.4‐61.6)	10.7 (1.7‐39.1)	13.7 (4.2‐48.2)
Missing	10	9	4	0
Glucose (mmol/L)	6.7 (2.8‐24.4)	6.4 (3.8‐18.3)	6.4 (2.9‐24.6)	5.6 (2.4‐13.2)
Missing	12	0	5	6

ALP, alkaline phosphatase; ALT, alanine transaminase; AMC, absolute monocyte count; ANC, absolute neutrophil count; AST, aspartate transaminase; CA19‐9, carbohydrate antigen 19‐9; ECOG, Eastern Cooperative Oncology Group; KPS, Karnofsky performance status; MPC, metastatic pancreatic cancer; PLT, platelet.

### 3.2 Prognostic factors for OS

As shown in Table 2, univariate Cox analysis revealed that 14 of 23 evaluated baseline characteristics were correlated with OS. The 14 baseline characteristics were performance status, liver metastasis, CA19‐9 log‐value, white blood cell (WBC), ANC, absolute monocyte count (AMC), platelet (PLT), albumin, protein, alanine transaminase (ALT), aspartate transaminase (AST), alkaline phosphatase (ALP), lactate dehydrogenase (LDH), and bilirubin. Furthermore, multivariate analysis showed that 6 factors including performance status, liver metastasis, CA19‐9 log‐value, ANC, PLT, and albumin were independent prognostic factors.

**Table 2:** Univariate and multivariate analysis of prognostic factors for OS in MPC patients

Characteristics	Univariate analysis			Multivariate analysis		
	HR	95% CI	*P*‐value	HR	95% CI	*P*‐value
Age (y, range)	1.003	0.992‐1.013	.629			
Gender						
Male	1.125	0.921‐1.376	.249			
Female	Reference					
Performance status						
ECOG PS = 0 (or KPS = 100)	0.263	0.161‐0.431	<.001	0.266	0.154‐0.457	<.001
ECOG PS = 1 (or KPS = 90 or 80)	0.515	0.333‐0.795	.003	0.517	0.323‐0.827	.006
ECOG PS = 2 (or KPS = 70)	Reference			Reference		
Body mass index (kg/m^2^)	0.988	0.965‐1.011	.308			
Primary tumor site						
Head and neck	0.988	0.798‐1.223	.909			
Body and tail	Reference					
Liver metastasis						
Yes	1.696	1.306‐2.202	<.001	1.491	1.114‐1.996	.007
No	Reference			Reference		
Lung metastasis						
Yes	1.065	0.876‐1.294	.529			
No	Reference					
CA19‐9 log‐value (U/mL)	1.195	1.107‐1.290	<.001	1.114	1.030‐1.206	.007
RBC (×10^12^/L)	0.849	0.689‐1.046	.133			
Hemoglobin (g/L)	0.993	0.987‐1.000	.057			
WBC (×10^9^/L)	1.096	1.064‐1.129	<.001	0.894	0.759‐1.053	.179
ALC (×10^9^/L)	0.923	0.778‐1.096	.360			
ANC (×10^9^/L)	1.120	1.084‐1.157	<.001	1.199	1.016‐1.415	.031
AMC (×10^9^/L)	1.991	1.438‐2.757	<.001	0.919	0.541‐1.563	.756
PLT (×10^9^/L)	1.001	1.001‐1.002	.001	1.001	1.000‐1.002	.021
Albumin (g/L)	0.966	0.952‐0.979	<.001	0.940	0.906‐0.975	.001
Protein (g/L)	0.974	0.957‐0.992	.005	0.997	0.972‐1.022	.791
ALT (IU/L)	1.004	1.001‐1.007	.006	1.002	0.996‐1.007	.500
AST (IU/L)	1.009	1.005‐1.013	<.001	1.000	0.990‐1.010	.992
ALP (U/L)	1.001	1.001‐1.002	<.001	1.000	0.999‐1.001	.720
LDH (U/L)	1.002	1.001‐1.002	<.001	1.001	1.000‐1.002	.152
Bilirubin (μmol/L)	1.027	1.012‐1.043	<.001	1.018	1.000‐1.036	.052
Glucose (mmol/L)	1.016	0.986‐1.046	.299			

ALP, alkaline phosphatase; ALT, alanine transaminase; AMC, absolute monocyte count; ANC, absolute neutrophil count; AST, aspartate transaminase; CA19‐9, carbohydrate antigen 19‐9; ECOG, Eastern Cooperative Oncology Group; KPS, Karnofsky performance status; LDH, lactate dehydrogenase; MPC, metastatic pancreatic cancer; OS, overall survival; PLT, platelet.

### 3.3 Establishment and internal validation of the nomogram

**Figure 2:**
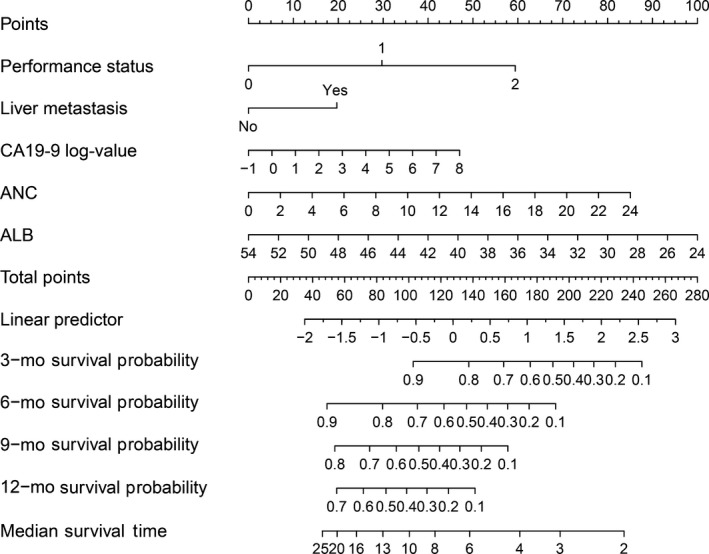
Prognostic nomogram for predicting 3‐, 6‐, 9‐, and 12‐mo overall survival probability based on performance status, liver metastasis, CA19‐9 log‐value, ANC, and albumin in patients with MPC. Each status of 5 independent factors has a corresponding value to the “Points” line at the top of the scale. Then the total point score was calculated by summing these 5 “Points” values. Based on the score, draw a downward vertical line from the “Total Points” line, predicting 3‐, 6‐, 9‐, and 12‐mo survival probability in a given patient. The Harrell's *C*‐index for the nomogram was .683. ANC, absolute neutrophil count; CA19‐9, carbohydrate antigen 19‐9; MPC, metastatic pancreatic cancer

**Figure 3:**
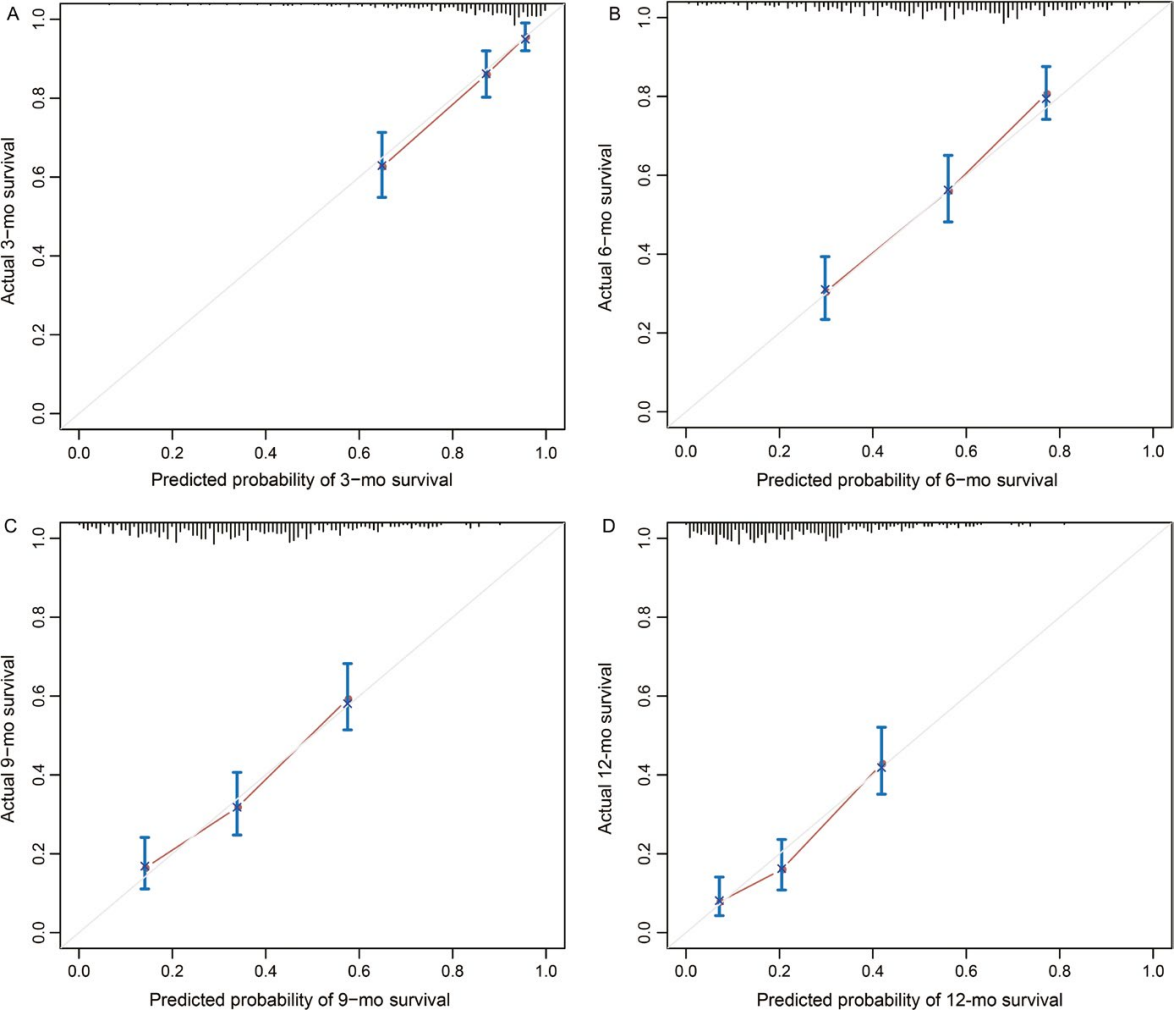
Calibration curves of the nomogram for the prediction of 3‐, 6‐, 9‐, and 12‐mo survival (A) 3 mo, (B) 6 mo, (C) 9 mo, and (D) 12 mo. The diagonal line: the ideal calibrated model. Black line: actual calibration. Circles: median. X: mean. 95% confidence intervals are depicted for each point along the calibration curve

As illustrated in Figure 2, a nomogram was established based on the results of Cox regression model, considering both clinical and statistical significance when selecting variables for inclusion.^17^ The nomogram predicted the median OS time and survival probabilities of MPC patients at 3‐month, 6‐month, 9‐month, and 12‐month. The *C*‐index for OS prediction was .683. After adjustment by bootstrapping with respective 1000 re‐samples, the calibration plots were illustrated for 3‐month, 6‐month, 9‐month, and 12‐month survival (Figure 3).

### 3.4 Prognostic score based on nomogram for OS stratification

**Figure 4:**
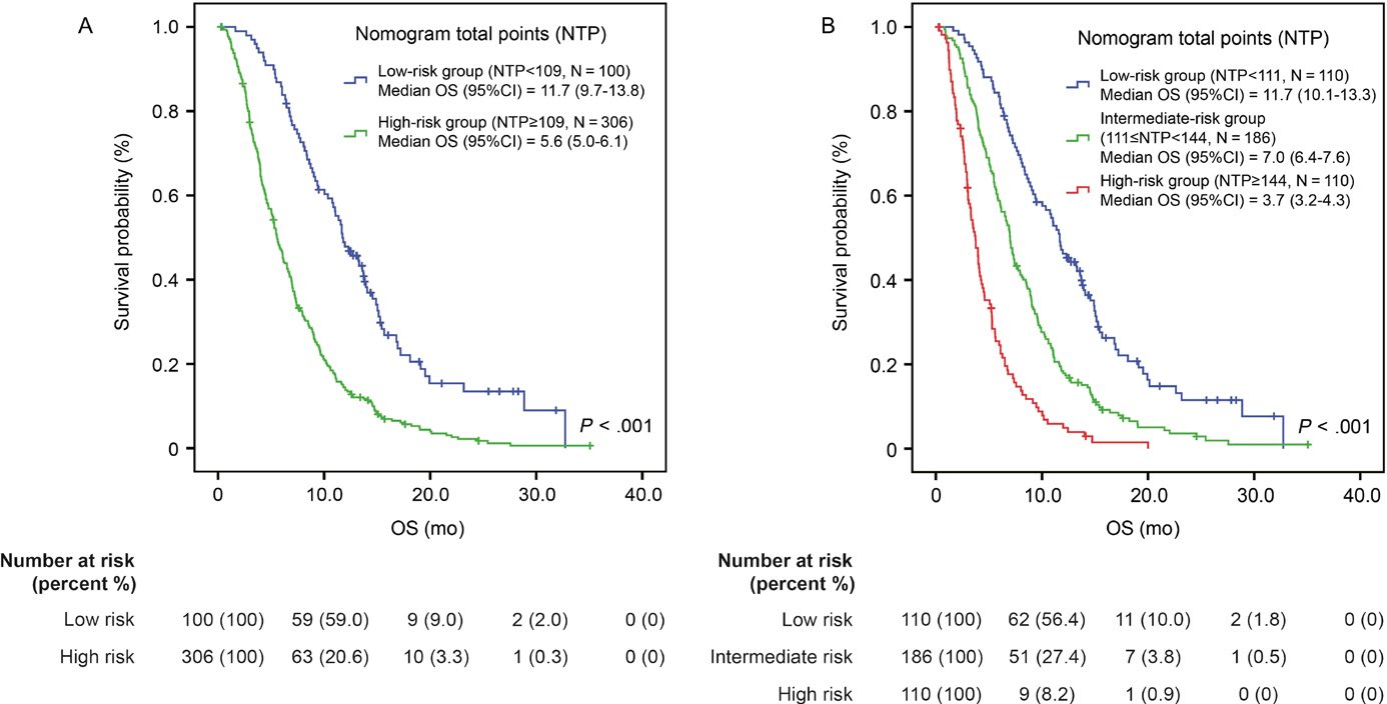
Kaplan‐Meier analysis according to NTP in the training cohort. A, 2‐risk groups; B, 3‐risk subgroups. NTP, nomogram total points

To give a reasonable stratification of OS, the patients were divided into 2 groups on the basis of an optimal cut‐off value of NTP identified by receiver operating characteristics (ROC) curve (Figure S1): a low‐risk group (NTP < 109, N = 100) and a high‐risk group (NTP ≥ 109, N = 306). The median OS of the low‐risk group and high‐risk group is 11.7 months (95% CI: 9.7‐13.8) and 5.6 months (95% CI: 5.0‐6.1; *P* < .001, Figure 4A), respectively. To give a more specific stratification for patients with high risk, the patients were further categorized into the following tertiles of risk: low‐risk group (NTP < 111, N = 110), intermediate‐risk group (111 ≤ NTP < 144, N = 186) and high‐risk group (NTP ≥ 144, N = 110; Figure 4B). The OS was significantly different among the 3 subgroups (*P* < .001). The median OS was 3.7 months (95% CI: 3.2‐4.3) in high‐risk group, 7.0 (95% CI: 6.4‐7.6) months in intermediate‐risk group and 11.7 (95% CI: 10.1‐13.3) months in low‐risk group, respectively.

### 3.5 External validation of the prognostic nomogram

**Figure 5:**
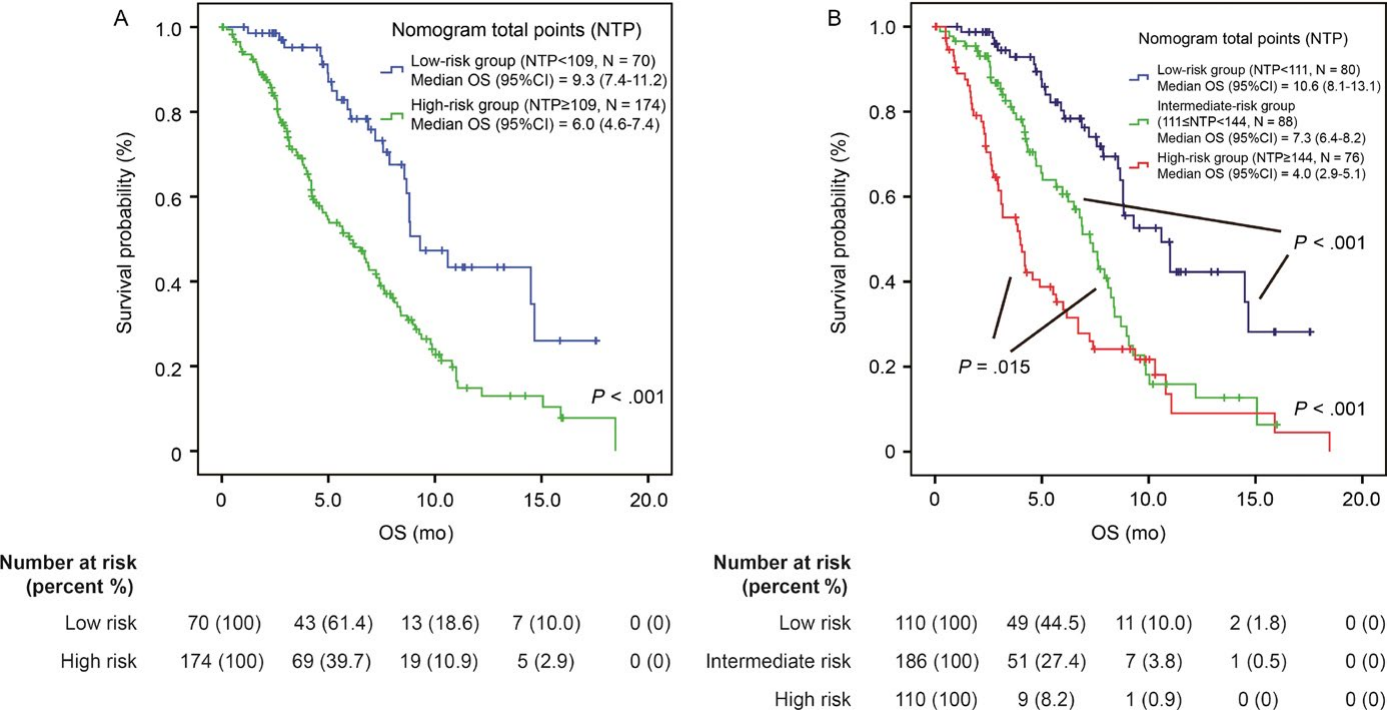
Kaplan‐Meier analysis according to NTP in the validation cohort. A, 2‐risk groups, B, 3‐risk subgroups. NTP, nomogram total points

Next, we further verified these results in 2 cohorts of external patient population from the comparator arm of the trial of NCT00574275 and real world study. Most of the patients in the validation cohort were Caucasian. As expected, the nomogram exhibited a good discernible ability (*C*‐index .699). The median OS was 9.3 months (95% CI: 7.4‐11.2) for the low‐risk group and 6.0 months (95% CI: 4.6‐7.4) for the high‐risk group (*P* < .001, Figure 5A). In the 3‐risk subgroup, the median OS was 10.6 (95% CI: 8.1‐13.1), 7.3 (95% CI: 6.4‐8.2) and 4.0 (95% CI: 2.9‐5.1) months for low, intermediate and high‐risk subgroups (*P* < .001, Figure 5B), respectively. In the testing cohort, the nomogram also showed an acceptable discernible ability (*C*‐index .658). The median OS was 9.7 months (95% CI: 5.3‐14.1) for the low‐risk group and 6.0 months (95% CI: 5.1‐6.9) for the high‐risk group (*P* = .012).

## 4 DISCUSSION

The prognosis of pancreatic cancer is affected by various factors. Currently, the lack of an efficient prognostic tool for OS in MPC leads to the difficulty in making decision on precise treatment. To resolve this issue, we used the data from 3 open clinical trials and real‐world study to establish and validate a prognostic nomogram based on clinicopathology characteristics. This model showed an acceptable discernibility and good calibration in predicting OS of MPC patients. Also, our results showed that patients with a lower NTP, a prognostic score derived from nomogram, had better OS than those with a higher NTP. The identification of different risk groups with different potential outcome may offer the opportunity to select risk‐adapted strategies for MPC. In addition, using this nomograms, patients with MPC can be evaluated and stratified in clinical trials to reduce heterogeneity among different treatment arms.^11^

Up to date, many biomarkers have suggested their prognostic value in patients with pancreatic cancer, but only a few of them are routinely used in clinical practice.^7^ The only biomarker approved by Food and Drug Administration (FDA) is CA19‐9, but approximately 5%‐10% of pancreatic cancer patients are Lewis negative, which means they do not express CA19‐9.^23^, ^24^, ^25^ Thus, there is a need in finding a group of biomarkers to predict comprehensively the prognosis for pancreatic cancer. In this study, we identified 6 independent prognostic factors, including performance status, liver metastasis, CA19‐9 log‐value, ANC, albumin, and PLT. All of them were previously reported for their prognostic value in pancreatic cancer in spite of controversial results. In our study, the former 5 factors were included into the nomogram based on clinical evidence and statistical significance.^26^, ^27^ Although the *P*‐value for PLT was .021, both 95% CI (1.000‐1.002) and β‐coefficient (.001) showed its limited effect on the OS when compared with the other 5 factors. Moreover, previous studies focused mainly on the prognostic value of PLT to lymphocyte ratio (PLR).^28^, ^29^

The predictive accuracy (usually measured via *C*‐index) and external applicability are always the 2 most important aspects for prognostic models. Intriguingly, the *C*‐index of our nomogram was .683, extremely close to those by Hamada et al (.686) and Vernerey et al (.68), but slightly higher than those reported by Brennan et al (.64) and Ferrone et al (.62). Notably, the former 2 nomograms were developed in patients with advanced (locally advanced or metastatic or both) pancreatic cancer while the latter 2 nomograms were developed in patients with stage I‐IV. Thus, it is conceivable that the accuracy of these nomograms will decreased when applied to patients with a larger heterogeneity. Unlike previous studies, our nomogram was validated externally in patients from another independent randomized controlled trial (RCT) and real‐world cohort. In the validation cohort and testing cohort, the nomogram also displayed a good applicability with a *C*‐index of .699 and .658, respectively. Furthermore, based on optimal cut‐off values of NTP identified by 2 different methods, patients with MPC in training cohort, validation cohort and testing cohort showed a considerable heterogeneity in their OS‐risk profiles with clearly different risk groups.

There are several advantages to this study. First, we developed the nomogram based on detailed data from 3 relatively larger international clinical trials (totally included 718 patients) and real‐world study (including 133 patients). Second, aside from the convincing source of the data, the nomogram was developed and validated strictly abiding by the recommended guide for nomograms.^16^, ^17^ Specifically, there were 4 steps in establishing this nomogram: definition of the enrolled subjects and outcome, specification for important variables, development and validation. Third, the accuracy of the nomogram was assessed in the training cohort by *C*‐index and calibration while its external applicability was evaluated in the validation cohort and testing cohort. The training cohort, validation cohort, and testing cohort were from independent population, suggesting the robustness of the results. Finally, the biomarkers included in the nomogram were noninvasive, easy to acquire and affordable for the patients, leading to a wide use in clinical practice.

Several limitations should be mentioned in this study. First, although a broad spectrum of covariates was included, other factors which might influence OS, such as C‐reactive protein (CRP),^30^ failed to be evaluated in this model. Second, emerging evidence demonstrates that the RCT condition does not correspond with the clinical real one.^31^ For example, over 90% of patients receiving gemcitabine monotherapy in 3 RCTs were with ECOG PS 0‐1 or KPS ≥ 80. However, in the clinical real condition, this kind of patients are often recommended to receive combined therapy such as fluorouracil‐leucovorin‐irinotecan‐oxaliplatin (FOLFIRINOX)^32^ or gemcitabine plus S‐1.^33^ Thus, it was reasonable that the predictive accuracy decreased when the nomogram was applied in the testing cohort, and a validation in a larger cohort of “real world” patients with MPC is still required to assess the external applicability of the nomogram.

In conclusion, the prognostic nomogram with NTP can predict OS for patients with MPC with considerable accuracy.

## CONFLICT OF INTEREST

None declared.
