# Supplementary material for: Prediction of overall survival for metastatic pancreatic cancer: Development and validation of a prognostic nomogram with data from open clinical trial and real‐world study
Source: Cancer Med. 2018 Jun 1;7(7):2974–84. doi: 10.1002/cam4.1573 (PMC6051216; doi:10.1002/cam4.1573)
Supplement: Supplementary file 1 [file CAM4-7-2974-s001.doc]

**Supplement**

**
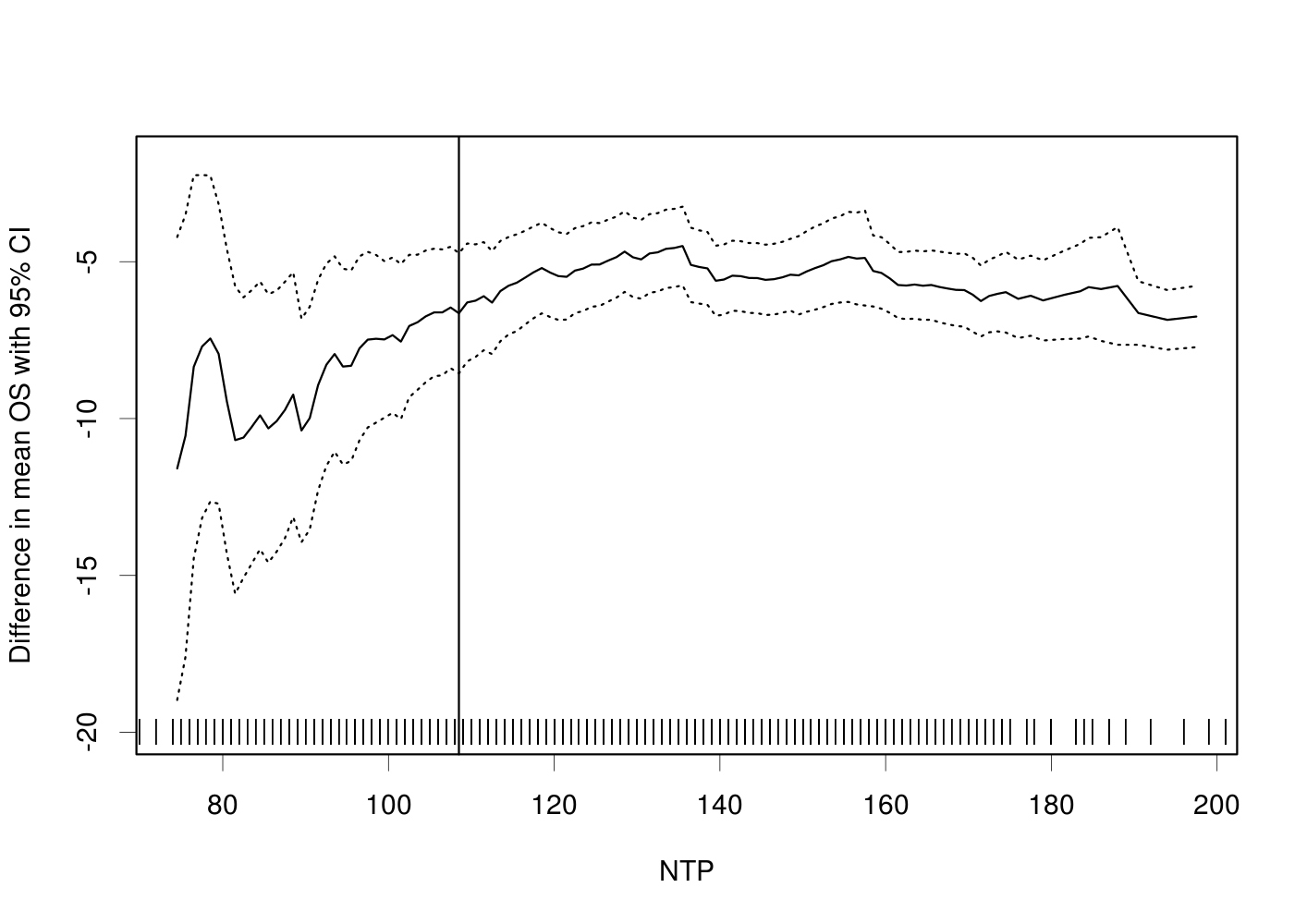
**

**Supplement Figure 1.** Hazard ratio (HR) for OS independent of cutoff point for NTP in patients with MPC. The vertical line designates the optimal cutoff point with the most significant (log-rank test) split. The plots were generated using Cutoff Finder.

| Supplement Table 1. Common prognostic models and biomarkers in pancreatic cancer | | | | | |
| --- | --- | --- | --- | --- | --- |
| Author | Year | Treatment | Prognostic markers | No. of patients | Models |
| Brennan et al. | 2004 | Resection | Age, Sex, Portal vein, Splenectomy, Margin of resection, Head.vs.others, Differentiation et al. | 555 | Nomogram |
| Ferrone et al. | 2005 | Resection | Age, Sex, Portal vein, Splenectomy, Margin of resection, Head.vs.others, Differentiation et al. | 424 | Nomogram |
| Hamada et al. | 2014 | Gemcitabine based chemotherapy | Age, Sex, ECOG PS, Tumour size, Lymph node Metastasis, Distant metastasis | 531 | Nomogram |
| Vernerey et al. | 2016 | Gemcitabine based chemotherapy | Age, Tumor size, Albumin, Pain, CA19-9 | 442 | Nomogram |
| Marechal et al. | 2007 | Gemcitabine based chemotherapy | KPS score, Weight loss (10%), AST | 99 | based on the regression coefficient |
| Kou et al. | 2016 | Gemcitabine based chemotherapy, S-1 | ECOG PS, Presence of distant metastatic disease, Recurrent or initially unresectable disease, CEA, CA19-9 and NLR | 306 | based on the regression coefficient |
| Yi et al. | 2011 | Gemcitabine based chemotherapy | CRP, Albumin, Liver metastasis, Ascites dissemination | 298 | based on the No. of prognostic factors |
| Xue et al. | 2015 | Gemcitabine based chemotherapy, S-1 | ECOG PS、CA19-9、CRP | 118 | based on the No. of prognostic factors |
